# Two Secondary Introductions From a Shared Bridgehead Population Show Evidence of Divergent and Parallel Selection

**DOI:** 10.1111/mec.70495

**Published:** 2026-07-29

**Authors:** Adi Nugroho, Sebastien Comte, Patrick Barrière, Robert B. Allen, William B. Sherwin, Lee A. Rollins

**Affiliations:** ^1^ Evolution & Ecology Research Centre UNSW Sydney Kensington New South Wales Australia; ^2^ Department of Bioresources Technology and Veterinary, School of Applied Sciences Gadjah Mada University Yogyakarta Indonesia; ^3^ Vertebrae Pest Research Unit, NSW Department of Primary Industries and Regional Development Orange New South Wales Australia; ^4^ Agence néo‐Calédonienne de la Biodiversité Koné New Caledonia; ^5^ Independent Researcher Lincoln New Zealand

**Keywords:** biological invasion, *Cervus timorensis*, parallel evolution, reduced‐representation sequencing, *Rusa timorensis*

## Abstract

Biological invasions offer large‐scale experiments for examining rapid evolution and testing the predictability of adaptive change. Assessing the repeatability of such adaptive responses, however, requires replicated introductions that share similar demographic and environmental contexts. Single introduction events are common during biological invasion, but replicated secondary introductions from a shared source are rare. Here, we take advantage of a unique study system involving the introduced rusa deer ( *Cervus timorensis* ) to investigate whether synchronous secondary introductions from a shared bridgehead population show similar evolutionary trajectories. We utilised 5298 DArTSeq Single Nucleotide Polymorphisms (SNPs) from a bridgehead population and two secondary populations to estimate genetic diversity, characterise population structure and detect SNPs putatively under selection. We found an apparent reduction in genetic diversity across serial introductions, consistent with strong founder effects. Population genetic analyses revealed two genetic clusters, indicating strong differentiation and independent evolutionary trajectories between secondary populations. A genome‐wide scan using BayPass identified outlier SNPs potentially involved in adaptive responses and some evidence of parallel selection, likely driven by common environmental pressures. We found candidate genes linked to the outlier loci that were associated with functions consistent with adaptive mechanisms. This study highlights that repeated introductions from a common source can further reduce genetic diversity and increase population differentiation, yet still produce parallel adaptive responses under similar environmental conditions.

## 1 Introduction

Biological invasions pose a significant threat to biodiversity (Allendorf and Lundquist 2003; Vantarová et al. 2023; Walker and Steffen 1997), causing the decline of native species (Mollot et al. 2017), disrupting ecosystem functions (Linders et al. 2019) and contributing to hybridisation with native species (Muhlfeld et al. 2014; Rhymer and Simberloff 1996). They also cause major economic impacts, resulting in global annual costs surpassing US$423 billion (IPBES 2023). Yet, populations of invasive species offer rare, empirical opportunities to examine evolution on large scales (Roux 2022; Sakai et al. 2001), which could inform the management of declining populations in their native range (Garzón‐Machado et al. 2012). For example, 92 tammar wallabies ( *Macropus eugenii* ) were reintroduced to their native range after genetic assessment confirmed that the source population originated from South Australia (Sharp et al. 2010; Taylor and Cooper 2006).

Introduced populations may encounter novel environments, including differences in climate, predation, competition and resource availability, which can lead to selection and local adaptation (Lee 2002). Rapid evolution, a common trait in invasive species (Rollins et al. 2013), is a key factor to their establishment success (Whitney and Gabler 2008). Genetic evidence for adaptation following introductions to novel environments is increasingly documented, including frequent detection of loci under selection (reviewed by Kolodziejczyk et al. 2025). That review included studies with both single and multiple introduction histories, highlighting that adaptation can be detected despite demographic processes such as bottlenecks and drift. However, evaluating whether such adaptive responses are repeatable requires replicated introductions from the same source population (McGaughran et al. 2025; Pearless and Freed 2024). When such replicated introductions occur into different environments, they can be used to test how ecological differences drive divergent evolution (Colauti and Barret 2013). On the other hand, if the introductions occur in similar environments, they can reveal which adaptations occur in parallel (de Jong et al. 2021; Stuart et al. 2017). Studies on replicated introductions are limited and, even when documented, invasion histories are often complex and difficult to reconstruct (Bock et al. 2015; Estoup and Guillemaud 2010).

**FIGURE 1:**
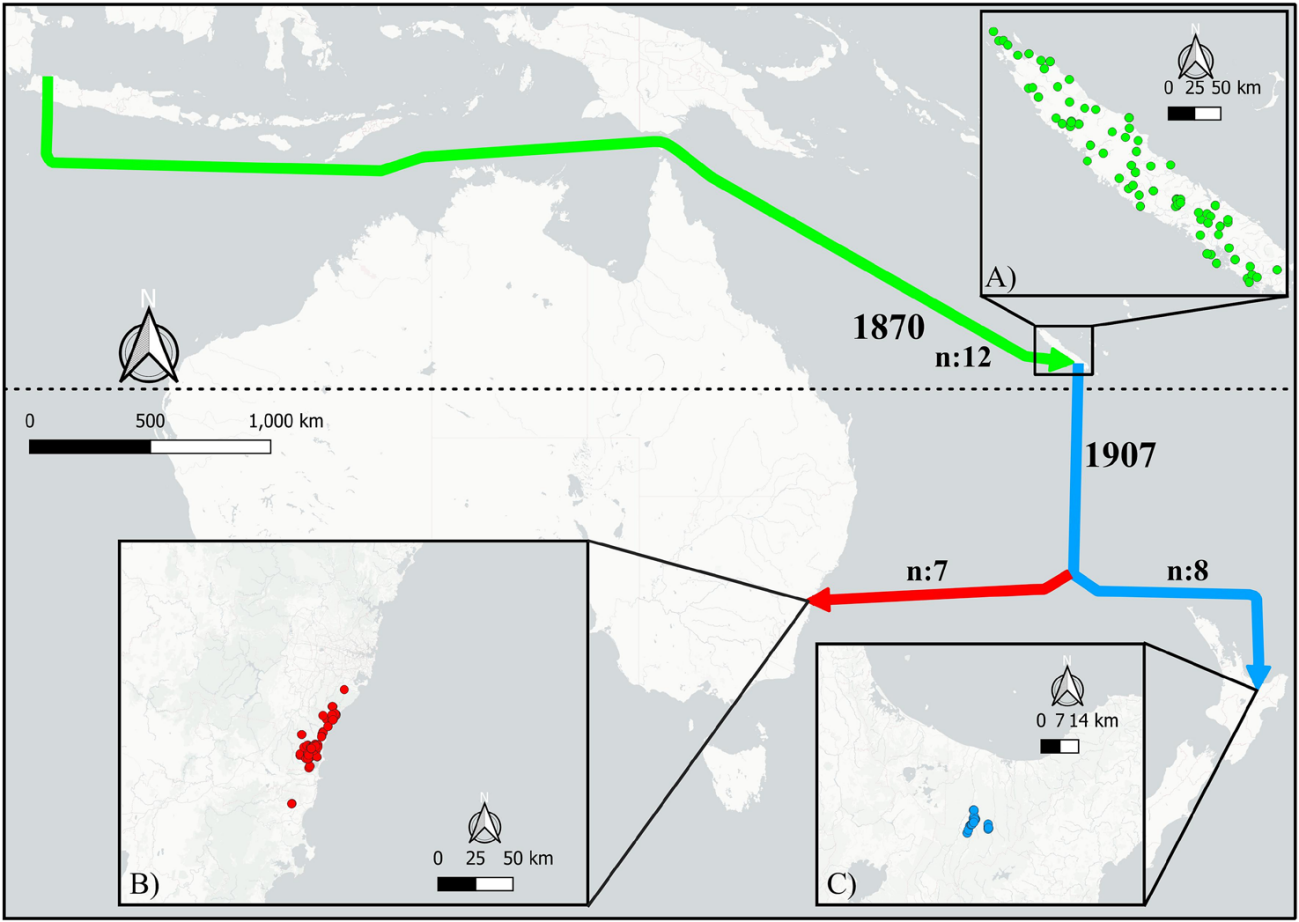
Rusa deer introduction route to Oceania. First, in 1870, rusa deer were introduced to New Caledonia. After a few generations, from this bridgehead population, they were subsequently introduced to Illawarra, Australia, and Te Urewera, New Zealand, in 1907. Dashed line indicates the tropic of Capricorn, dividing the tropic zone in the north and the temperate zone in the south. n indicates number of founders, while insets A, B, and C show sample distribution in New Caledonia, Australian Illawarra, and New Zealand Te Urewera, respectively.

The introduction of rusa deer ( *Cervus timorensis* ; nomenclature follows Jackson et al. 2015; synonym: *Rusa timorensis*) into Oceania provides a unique opportunity to study multiple introductions to similar environments (Figure 1). Native to the islands of Java and Bali, Indonesia, rusa deer have been introduced across Oceania (Hedges et al. 2015). Historical records indicate that 12 founders (eight females and four males) were first introduced to New Caledonia in 1870 (Allen 2021; de Garine‐Wichatitsky and Roques‐Rogery 2005), resulting in the establishment of the largest population in the world, estimated at 250,000–370,000 individuals in 2011 (Comité français de l'UICN, O 2011; Fort and Barrière 2021). This population has a local density greater than 100 deer/km^2^ (Witczuk et al. 2025), even in mountainous areas (Alliod and Cherif 2024), and had colonised the whole of the main Grande Terre island in less than 30 years (Desvals and Devinck 1991). As a result, they pose a significant threat to biodiversity by overgrazing native flora and to soil conservation by trampling and destroying the forest understory (de Garine‐Wichatitsky et al. 2005; Tramier et al. 2021). Although this population has become highly valued as a source of red meat and hunting prestige (Nugent et al. 2025), control of this population is now a major priority (CEN Nouvelle‐Calédonie 2021) in a region recognised as one of the world's most significant biodiversity hotspots (Myers 1988; Myers et al. 2000).

In 1907, eight individuals from this seasonal tropical population in New Caledonia were relocated to Te Urewera on the North Island of New Zealand (Allen 2021) and seven individuals were relocated to the Illawarra region on the east coast of Australia (Long 2003). Consistent with the bridgehead effect (i.e., serial introduction) (Lombaert et al. 2010) both secondary introductions resulted in successful establishment of rusa deer populations in temperate climates, reaching several thousand individuals in New Zealand by 2021 (Allen 2021) and an estimated 7000 in the Illawarra by 2004 (Moriarty 2004b). In Te Urewera New Zealand, rusa deer affect vegetation structure and composition by browsing native plants (Allen et al. 1984), whereas in Illawarra Australia, impacts of rusa deer include vehicle collisions, property damage and native species browsing (Dawson 2016). If the Australian Illawarra and New Zealand Te Urewera populations have adapted to their new environments, these two founder populations may have undergone parallel evolution. Both populations have encountered similar climate conditions and thus have potentially been subjected to similar selection regimes. Although differences in hunting pressure may influence the evolution of these populations, we did not include this factor because the available hunting records in the two populations differ in temporal coverage and number of records (Allen et al. 2015; Comte et al. 2024; Forsyth et al. 2011; Nugent 1992), making direct comparison unreliable.

Investigating the presence of parallel evolution is critical for understanding whether adaptation follows predictable pathways under shared selective regimes or primarily reflects demographic or stochastic factors. Evidence of parallel evolution of introduced populations from a common ancestor has been documented (de Jong et al. 2021; Sherpa et al. 2026; Stuart et al. 2022). Using selection analyses, those studies show that independent introductions can exhibit repeated genetic responses to similar environmental conditions. However, not all of those replicated introductions occurred simultaneously. Similar timing of introductions is ideal because then the opportunity for evolution cannot be differentially affected by time since introduction.

In this study, we take advantage of this rare, large‐scale experiment to examine whether two synchronous secondary introductions from a shared bridgehead population show similar evolutionary trajectories following introduction. Specifically, we test three predictions: (1) that founder effects led to significantly reduced genetic diversity and increased population differentiation across rusa deer introduced populations, (2) that environmental differences among the bridgehead and secondary populations led to different genomic signatures of selection, potentially indicating local adaptation and (3) that the Illawarra, Australia and Te Urewera, New Zealand populations have undergone parallel evolution in response to similar climate conditions. This study provides rare empirical data highlighting how species respond to novel environments following introduction and revealing the evolutionary processes that may explain the establishment and spread of invasive species.

## 2 Methods

### 2.1 Sample Collection

New Caledonia, New Zealand, and Australia are separated by the South Pacific Ocean. The distance between New Caledonia and Illawarra, Australia is approximately 2009 km, and the distance from New Caledonia to Te Urewera, New Zealand is 2168 km. Sampling sites in New Caledonia were broadly distributed across the rusa deer's range of 17,000 km^2^, on the main island Grande Terre, where there is a mountain chain with tropical rainforest and peaks exceeding 1600 m (Moron et al. 2015), and additional small samples from peripheral islets. The mean total annual rainfall is 1100 mm (Vincent et al. 2007) and an average temperature of 23.5°C (Fosting 2021), ranging from 18.1°C in the cool season to an average of 26.8°C in the warm season (Moron et al. 2015). In Te Urewera, New Zealand, rusa deer occupied 2928 km^2^ (Allen 2021). Sampling sites in this region occur in a mountainous area with daily precipitation of 2.2 ± 0.4 mm during winter and 3.4 ± 1.0 mm during summer, while the mean winter temperature was 7.4°C and the mean summer temperature was 17.2°C in 2023 (Allen et al. 2015; NIWA 2024). In the Illawarra region of Australia, rusa deer approximately occupied 2800 km^2^ area (Crittle 2023) where there is an escarpment with a steep outcrop ranging from 300 to 700 m above sea level (NSW Office of Environment and Heritage 2014). The annual rainfall is 1500–1600 mm, while the mean summer temperature is 18°C–22°C, and the temperature in the winter is 12°C–14°C (NSW Office of Environment and Heritage 2014).

We collected tissue samples across New Caledonia during management activities and subsistence hunting in 2009, 2010 and 2023 (*n* = 159; 60 samples stored in Allflex Tissue Sampling Units, 6 in alcohol and 93 without preservative), and opportunistically in Te Urewera, New Zealand, during recreational hunting in 2023 (*n* = 29, all stored in Allflex Tissue Sampling Units) (Figure 1). We extracted DNA using a Puregene Tissue Kit (Qiagen) following the manufacturer's instructions and quantified the extracted DNA using a Qubit Fluorometric Quantification kit (Thermo Fisher Scientific). We checked a subset of genomic DNA (gDNA) for the presence of nucleases by incubating 2 μL of extracted DNA in 1× restriction enzyme buffer at 37°C for 2 h.

We chose to use Diversity Array Technology Pty Ltd. (Canberra, Australia) for reduced‐representation sequencing (DArTSeq). Reduced‐representation sequencing is relatively cost‐effective and does not depend on a species‐specific reference genome (Allendorf et al. 2022); therefore, it is suitable for non‐model species like rusa deer. DArTSeq targets a reduced fraction of the genome by using methylation‐sensitive restriction enzymes (here, *Pstl* and *Sphl*) to reduce complexity and to filter out repetitive regions of the genome (Kilian et al. 2012), a benefit of this methodology over other forms of reduced representation sequencing. After adapter ligation, libraries are sequenced using Illumina NovaSeq X.

We submitted 188 rusa deer gDNA extracts for medium‐density DArTSeq (single‐end reads). Nine DNA samples could not be successfully sequenced, resulting in a data set containing 150 samples from New Caledonia and 29 samples from New Zealand. In addition to this, we incorporated DArTSeq data produced using the same protocol into our analyses from 167 rusa deer samples from the Illawarra region of Australia (Li‐Williams et al. 2023). In total, we analysed 346 rusa deer sequences.

### 2.2 Variant Calling

We employed the computational cluster at the University of New South Wales (PVC Research Infrastructure, UNSW Sydney 2010) to analyse all sequence data described above, including the reanalysis of raw sequences from Li‐Williams et al. (2023). A reference‐based variant calling approach was used to analyse fastq files using STACKS v2.2 (Catchen et al. 2013). We used the process_radtags function to clean and remove low‐quality raw reads. We employed fastp/0.23.4 (Chen et al. 2018) to filter quality (‐q 22), trim poly‐G tails (*‐‐trim_poly_g*), remove adapter sequence (*‐‐adapter_sequence*), trim reads to 69 bp (*‐‐b 69*), and discard reads shorter than 69 bp (*‐‐length_required 69*). We then used the Burrows‐Wheeler Aligner (bwa/0.7.17) (Li and Durbin 2009) to align reads to the closest deer genome available, the hog deer ( *Axis porcinus* ) reference genome (GCA_003798545.1). Based on the quality control with Fastqc v0.11.9 (Andrews 2010), we removed the first five nucleotides of each read (‘*‐B 5*’ command).

We conducted variant calling using the *populations* program in STACKS and employed initial quality filtering with a minimum loci log likelihood value of 10 (–‐lnl_lim ‐10). We kept one random SNP per locus (*–write_random_snp*). Afterwards, we performed several filtering steps with vcftools v0.1.14 (Danecek et al. 2011). We filtered the dataset by applying a minimum read depth of 2 (‐minDP 2), a maximum read depth of 50 (*‐maxDP 50*), and a minimum genotype quality of 15 (*‐minGQ 15*). We then removed missing data (*‐‐max‐missing 0.2* ) and excluded individuals with more than 70% missing genotypes. This process led to the removal of 46 samples from New Caledonia due to high missingness. Most of these excluded samples had not been preserved, indicating that the sample preservation contributed to the amount of missing data. We excluded rare variants with a minor frequency below 0.05 (*‐‐maf 0.05*) and applied a stricter SNP‐level filter (*‐‐max‐missing 0.5*) to retain higher‐quality variants for downstream analyses. This resulted a final dataset containing 5298 SNPs across 300 individuals (New Caledonia, *n* = 104; New Zealand, *n* = 29, Australia, *n* = 167).

### 2.3 Population Structure and Genetic Diversity

We estimated genetic diversity metrics using the gl.report.heterozygosity function in dartR V2.9.9.5 (Gruber et al. 2018) implemented in R V4.3.3 (R Core Team 2025), which calculates the number of polymorphic loci and unbiased expected heterozygosity (*uHe*). We visualised population structure using Principal Component Analysis (PCA), performed with the gl.pcoa function in dartR. Using function gl.fst.pop in dartR, we measured genetic differentiation (*F*_*ST*_) between the three regions. To infer ancestry proportions and to determine whether the sampled individuals grouped into distinct genetic lineages, we employed ADMIXTURE v.1.3.0 (Alexander et al. 2009). In this program, we set a random seed based on the current time (‘‐s time’), explored hypothesised number of distinct populations (*K* values) ranging from 1 to 10 to allow detection of potential cryptic population structure, and computed cross‐validation error for each *K* (‘‐cv’). In addition, we explored the presence of genetic structure in New Caledonia and Te Urewera, New Zealand using PCA using gl.pcoa function in dartR. Population structure within Illawarra, Australia was previously described in Li‐Williams et al. (2023).

### 2.4 Detecting Signatures of Selection

To detect loci under selection and test their association with environmental covariates, we used BayPass v2.41 (Gautier 2015), which models allele frequency covariance among populations. We ran BayPass using the core model (*g_baypass*) with default parameters (pilot = 20, burn‐in = 5000 steps) to estimate population structure (Ω matrix) and calculate the *XtX* statistic for each SNP. We estimated a pseudo‐observed dataset (PODs) using the *simulate.baypass* () function in R, incorporating the estimated Ω matrix, sample size, and beta parameters from the real data. SNPs with *XtX* values more than the 99% threshold generated from PODs were considered indicative of adaptive divergence.

We then used the BayPass covariate model to examine associations between allele frequencies at these outlier SNPs and environmental variables by estimating Bayes Factors, allowing us to identify SNPs potentially involved in local adaptation. Population‐specific environmental variables (described below) were used in the BayPass auxiliary model. SNPs with high Bayes Factors (BF > 20 dB, defined as 10 × log_10_(BF)) were considered strongly associated with environmental variables, suggesting that these loci may play a role in adaptation. Outlier SNPs from this covariate analysis were intersected with those previously identified as *XtX* outliers using the core model above. SNPs appearing in both lists were classified as double outliers, meaning loci with both high population differentiation and a strong association with environmental variables.

We focused on environmental variables that showed contrast between the native range (low seasonal tropical) and the bridgehead population (seasonal tropical) and the two secondary introductions (temperate). These included Isothermality (Bio3), Temperature Seasonality (Bio4), Minimum Temperature of Coldest Month (Bio6), and Temperature Annual Range (Bio7). We downloaded these data from Worldclim (Fick and Hijmans 2017) at a resolution of ~1 km using the geodata packages (Hijmans 2026) in R. We tested collinearity between predictor variables using the *findCorrelation* () function from the caret package. All four variables were highly correlated (*r* > 0.7), so we retained Bio4 because it showed the highest differentiation between the native and introduced ranges (Figure S1). In addition to climate variability, we also used the Normalised Difference Vegetation Index (NDVI) as a proxy for habitat cover and food availability. We retrieved and calculated NDVI values across sampling points from Sentinel‐2 data using the Copernicus Data Space Ecosystem Browser (https://browser.dataspace.copernicus.eu/) (Figure S2). The BayPass covariate model expects one value of environmental data per population; therefore, we calculated the mean across all sampling points in each population.

Because both Australian Illawarra and New Zealand Te Urewera populations are situated in temperate regions, we predicted that there would be SNPs from these populations that were evolving in parallel. To detect SNPs potentially under parallel selection, we used the BayPass core model to perform pairwise comparisons between the New Caledonia‐New Zealand Te Urewera and New Caledonia‐Australian Illawarra populations. SNPs identified as outliers in both pairwise comparisons were considered candidates for parallel selection. We used a one‐sided hypergeometric test to determine whether the shared outliers were more frequent than expected by chance (Fury et al. 2006).

We annotated all the outlier SNPs to explore function and pathways. To do this, we extracted sequences surrounding the outlier SNPs and blasted them on the NCBI database. We retrieved gene names with the best hit matches (smallest e‐value and most similar to rusa deer) and matched the functional descriptions of the genes to Uniprot IDs. Once we retrieved gene names, we performed gene ontology (GO) enrichment analyses using the DAVID bioinformatics web‐based GUI (Sherman et al. 2022). We uploaded candidate genes to DAVID using ‘OFFICIAL_GENE_SYMBOL’ as the identifier type and performed GO enrichment analyses using *Mus musculus* as the reference species. Significance of enriched GO terms was evaluated using False Discovery Rate (FDR) correction for multiple testing, also in DAVID bioinformatics web‐based GUI (Sherman et al. 2022).

## 3 Results

### 3.1 Genetic Diversity

Consistent with our prediction, both populations from secondary introductions (Illawarra, Australia and Te Urewera, New Zealand) exhibited lower unbiased expected heterozygosity and a lower proportion of polymorphic loci than the bridgehead population of New Caledonia (Table 1).

**TABLE 1:** Genetic diversity from three populations of introduced rusa deer in Oceania. Abbreviations: n.Ind, the number of individuals without missing data; n.Loc, the number of loci that were present and were used to estimate heterozygosity; polyLoc, the number of polymorphic loci; monoLoc, the number of monomorphic loci; uHe, unbiased expected heterozygosity.

Population	n.Ind	n.Loc	polyLoc (% of total loci)	monoLoc (% of total loci)	uHe (± SE)
New Caledonia	69	5287	5219 (98.7%)	68 (1.3%)	0.364 (±0.002)
Illawarra, Australia	150	5298	4248 (80.2%)	1050 (19.8%)	0.255 (± 0.003)
Te Urewera, New Zealand	18	5023	4415 (87.9%)	608 (12.1%)	0.296 (±0.003)

The Australian Illawarra population showed strong differentiation from both the source population (*F*_ST_ = 0.20, 95% CI: 0.19–0.20) and the New Zealand Te Urewera population (*F*_ST_ = 0.28, 95% CI: 0.26–0.29). In contrast, the New Zealand Te Urewera population showed lower differentiation from the source population (*F*_ST_ = 0.06, 95% CI: 0.06–0.07).

### 3.2 Population Structure

**FIGURE 2:**
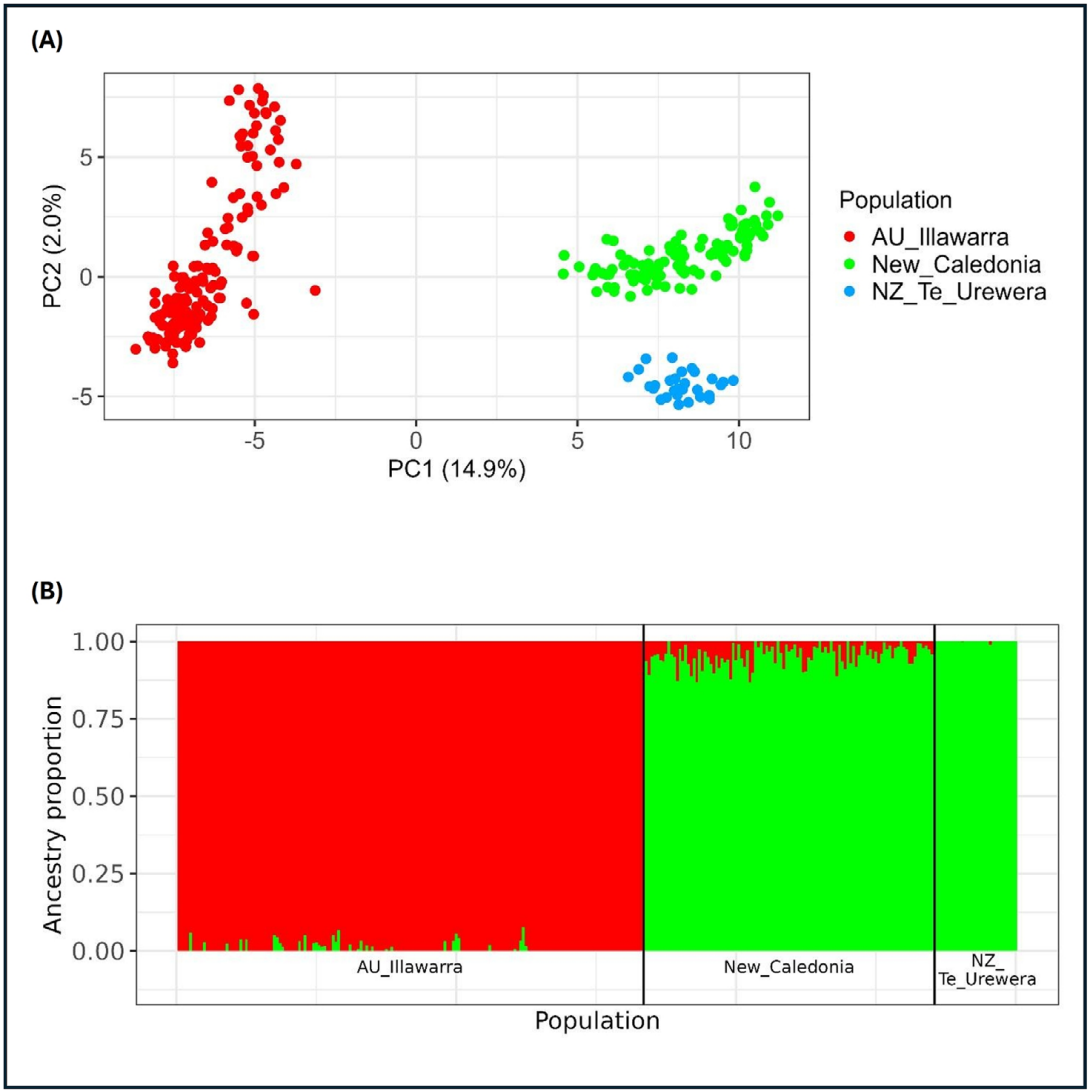
PCA (A) and structure plot (B) of the introduced rusa deer across Oceania.

We observe distinct genetic structure across the introduced rusa deer populations (Figure 2A). There is clear separation between the Australian Illawarra and New Caledonia/New Zealand Te Urewera populations (Figure 2A, PC1 explains 14.9% of the variation), and a less pronounced differentiation between the New Caledonia and New Zealand populations (PC2 explains 2.0% of the variation).

The ADMIXTURE analysis indicates the best‐supported population structure is *K* = 2 (Figure 2B, Figure S3). The Australian Illawarra formed a single cluster, while New Caledonia and New Zealand formed a second cluster. The low levels of admixture between these groups indicate a distinct evolutionary history of the Australian Illawarra population following introduction.

**FIGURE 3:**
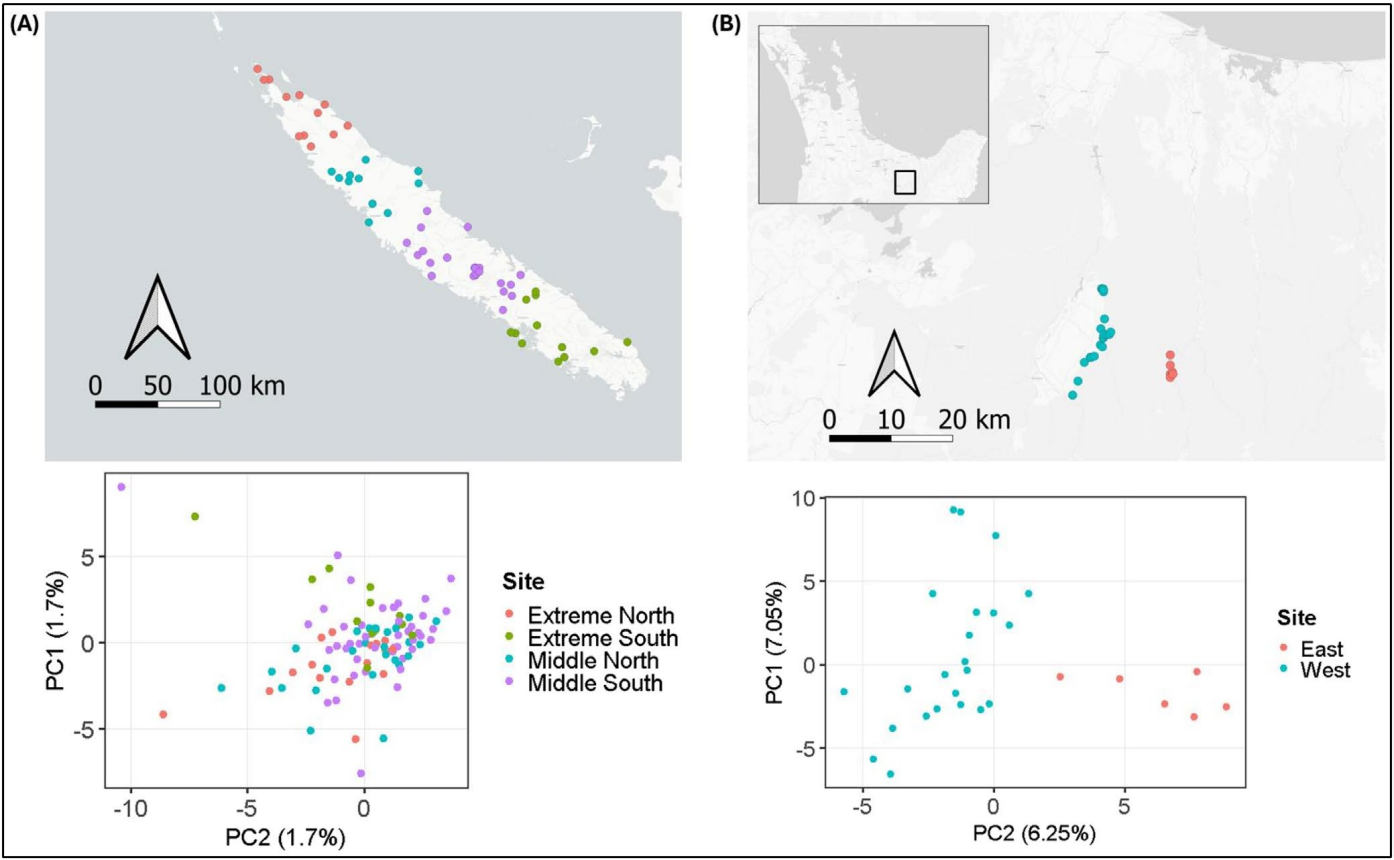
Sample locations (maps) and within‐population genetic structure (PCA) in the New Caledonia (A) and Te Urewera, New Zealand (B) rusa deer populations. The population genetic structure of the Illawarra, Australia populations is shown in Li‐Williams et al. (2023; Figure 2).

Within New Caledonia, individuals showed no discrete clustering among sampling sites (Figure 3A). We observed some within‐population genetic structure in New Zealand where PC2 explained 6.25% of the variance (Figure 3B, PC2). Population sub‐structure was previously identified in the Australian Illawarra introduction (two genetic groups; Li‐Williams et al. 2023).

### 3.3 Selection Analysis

**FIGURE 4:**
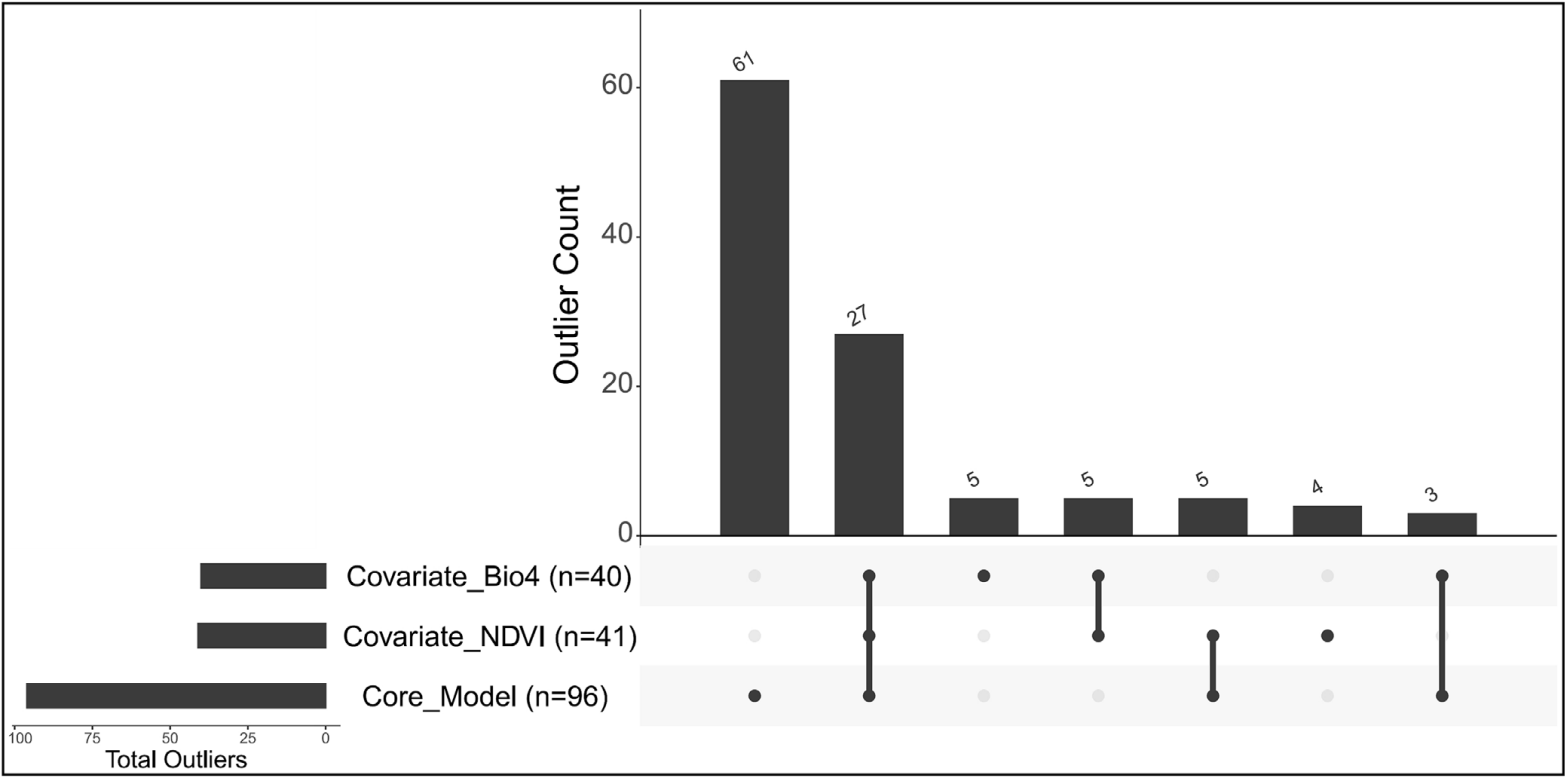
UpSet plot shows the abundance of outlier SNPs identified using each model (BayPass core model, Bio4 covariate model, NDVI covariate model) and their intersection. The horizontal bars on the left represent the total number of SNPs identified by each model. The vertical bars at the top indicate the number of outlier SNPs identified in only one model (indicated by single dots below *x*‐axis) and those identified across all combinations of models (indicated by multiple dots below *x*‐axis).

To identify loci potentially involved in local adaptation, we conducted three separate BayPass analyses: one using the core model (*XtX*), and two auxiliary models testing associations with environmental variables. The latter included Temperature Seasonality (Bio4) and Normalised Difference Vegetation Index (NDVI). The BayPass core model identified 96 outlier SNPs across the three populations, while the Bio4 and NDVI covariate model detected 40 (Figure S4) and 41 (Figure S5) outlier SNPs, respectively (Figure 4). Sharing of core model outliers SNPs with the outlier SNPs from either or both of the Bio4 covariate model and the NDVI covariate model indicates that they are strong candidates for local adaptation because they were identified in both the core and the environmental analyses (Figure 4).

Of the 96 outlier SNPs identified using the BayPass core model, the NCBI BLAST search returned 43 genes (Table S1). Gene ontology enrichment analysis for these 43 candidate genes identified one significantly enriched GO term, ‘regulation of kinase activity’ (*p* value < 0.05, FDR = 0.001). This GO term includes three genes containing outlier SNPs: *Xylosys and glucuronyltransferase* (*LARGE1*), *Protein NLRC5* (*NLRC5*) and *microcephalin* (*MCPH1*). There were no other GO terms that remained significant after FDR correction.

Of the 30 outlier SNPs shared between the BayPass core model and the Bio4 covariate model, and 32 outlier SNPs shared with the NDVI covariate model, we identified 11 and 12 annotated genes, respectively (Table 2; see Tables S2 and S3 for detailed gene functions). Ten genes were common to both intersections. The GO enrichment analysis did not identify any functional categories significantly overrepresented among the intersecting candidate genes.

**TABLE 2:** Genes annotations for SNPs detected as outliers by the Baypass core and Bio4 covariate models (Core_CovBio4), or by the Baypass core and NDVI covariate models (Core_CovNDVI).

Test	Locus ID	Gene name	Gene abbreviation	*e* Value	NCBI reference	Function
Core_CovBio4, Core_CovNDVI	18673	Heterogeneous nuclear ribonucleoprotein A3	*HNRNPA3*	5.00E‐23	XM_043894868.1	mRNA processing; mRNA splicing
Core_CovBio4, Core_CovNDVI	545302	Divergent protein kinase domain 1B	*DIPK1B*	5.00E‐23	XM_043916422.1	Enables protein binding
Core_CovBio4, Core_CovNDVI	612149	FUN14 domain‐containing protein 2	*FUNDC2*	8.00E‐21	XM_070366178.1	Transcription; Transcription regulation
Core_CovNDVI	755491	Death‐associated protein kinase 2	*DAPK2*	2.00E‐15	XM_043471711.1	Enables nucleotide binding; enables protein kinase activity
Core_CovBio4, Core_CovNDVI	29668	Beta‐sarcoglycan	*SGCB*	7.00E‐15	XM_065914046.1	Muscle organ development
Core_CovBio4, Core_CovNDVI	28099	Low‐density lipoprotein receptor‐related protein 1B	*LRP1B*	8.00E‐15	PQ075824.1	Endocytosis
Core_CovNDVI	155418	Gap junction gamma‐1 protein	*GJC1*	3.00E‐14	XM_019981967.2	Cell development; cell–cell signalling
Core_CovBio4, Core_CovNDVI	873768	leukocyte immunoglobulin‐like receptor	*LILR*	4.00E‐12	JX848345.1	Cell surface receptor signalling pathway
Core_CovBio4, Core_CovNDVI	690539	Immediate early response gene 2 protein	*IER2*	7.00E‐09	XR_009600604.1	Enables DNA binding; enables protein binding
Core_CovBio4, Core_CovNDVI	872835	D‐aminoacyl‐tRNA deacylase 2	*DTD2*	3.00E‐07	XM_019983496.2	Aminoacyl‐tRNA metabolism is involved in translational fidelity
Core_CovBio4, Core_CovNDVI	45263	Shieldin complex subunit 2	*SHLD2*	5.00E‐05	XM_019954105.2	DNA damage; DNA repair
Core_CovBio4	379754	Tetratricopeptide repeat protein 31	*TTC31*	5.00E‐04	XR_006543203.2	Protein binding
Core_CovBio4, Core_CovNDVI	85602	Proline‐rich protein 36	*PRR36*	2.00E‐03	XM_057708803.1	Encodes a large protein of unknown function that contains internal regions of low complexity sequence

We found 47 outliers from the core model shared between New Caledonia and Australia and 83 shared outliers between New Caledonia and New Zealand. Six of these outliers were shared between the two groups, a significantly greater overlap than expected by chance (one‐sided hypergeometric test, *p* < 0.01), indicating putative parallel evolution. Five of these six outliers were annotated: Growth Arrest Specific 2 (*GAS2*); REV*3*‐like, DNA directed polymerase zeta catalytic subunit (*REV3L*); Solute Carrier Family 19 Member 1 (*SLC19A1*); RCC1 and BTB domain containing protein 1 (*RCBTB1*); and Upstream Transcription Factor Family Member 3 (*USF3*) (Table S4). Functional enrichment analysis revealed that these genes are not significantly overrepresented in any known biological pathways.

## 4 Discussion

Our study highlights that even recently introduced populations from the same bridgehead source exhibit signatures of selection suggestive of local adaptation. Rusa deer were introduced to New Caledonia in 1870 and subsequently to Australian Illawarra and New Zealand in 1907. Given a generation time of 5 years (Hedges et al. 2015), these populations have experienced approximately 20 generations since their introduction to Illawarra, Australia and Te Urewera, New Zealand. A similar pattern of selection in comparable environments has been reported in recently introduced cervid populations originating from similar source populations (de Jong et al. 2021; Fuller et al. 2020). Moreover, the present study also provides key insights into the repeatability of the adaptive process by detecting evidence of parallel evolution among independently introduced populations, but also identifying introduction‐specific signals of selection.

### 4.1 Reduced Genetic Diversity and Population Differentiation

Historical records indicate that the founders of the Australian Illawarra and New Zealand Te Urewera were sourced and transported from New Caledonia on the same vessel (Long 2003). This secondary founding from an already bottlenecked New Caledonian population (de Garine‐Wichatitsky et al. 2009) caused us to predict further loss of genetic diversity in Australian Illawarra and New Zealand populations. Indeed, we found reduced genetic diversity following secondary introductions, a pattern supported by the widely held expectation of diversity loss through serial founder events (Clegg et al. 2001).

The observed divergence of the Australian Illawarra from the bridgehead population is consistent with founder effects followed by strong genetic drift, given only seven individuals were introduced as founders (Long 2003). In contrast, the Te Urewera, New Zealand population, which started with a similar number of introduced deer, did not show strong genetic differentiation from the bridgehead population. This could have resulted from the Te Urewera New Zealand population having higher levels of demographic expansion in the first few generations than did the Illawarra Australia population, despite the latter's higher contemporary population size. It is also possible that the Illawarra Australia founders were subdivided following introduction and that some lineages went extinct. Sex ratios might have been skewed in the Australian Illawarra founding population, impacting its effective population size and thus accentuating genetic drift. Unfortunately, the information available on the founding events is sparse, and with uncertain accuracy, it would be purely speculative to make further inferences.

It is also important to note that there may be low levels of gene flow into the Illawarra region in Australia. In addition to the Illawarra region, rusa deer were also introduced from various sources to several other locations in Australia, including Queensland, the Northen Territory and Victoria where they are now established (Moriarty 2004a). Illegal translocations of these individuals into the Illawarra region are possible (Moriarty 2004a), and therefore the Illawarra populations may have multiple rusa deer lineages. Furthermore, a recent study identified hybrids (7 of 172 individuals) between rusa and sambar deer in the Illawarra region (Hill et al. 2023), which could indicate that the population sub‐structure in that region is influenced by the introgression of sambar genetic material.

The within‐population genetic structure analyses revealed no evidence of structure in New Caledonia, suggesting a single panmictic population exists across the island, concordant with a previous microsatellite study of 628 rusa deer sampled across Grande Terre, New Caledonia, that found no genetic structure and the absence of dispersal barriers in the landscape (Frantz et al. 2023). Panmixia implies extensive dispersal and interbreeding of individuals across the 17,000 km^2^ mainland of New Caledonia. In a lowland area of this region, home range of rusa deer have been estimated to be only 498 ha for females and 1230 ha for males (Spaggiari and de Garine‐Wichatitsky 2006), but ongoing deer farming activities initiated in the 1980's (Garine‐Wichatitsky 2004) may facilitate translocations, with escapes and/or releases from captivity potentially contributing to widespread gene flow. From a management perspective, the presence of a single panmictic population could suggest that localised control efforts alone would be less likely to achieve a long‐term population suppression. A single panmictic population indicates high connectivity. Therefore, if rusa deer were culled in one location, individuals from surrounding areas could quickly recolonise the area. While large‐scale, coordinated management may be logistically and economically extremely challenging, prioritising control of hinds and fawns, and within areas with high value environments (e.g., reserve areas or national parks) may represent a more urgent, effective and feasible strategy.

In New Zealand Te Urewera, we identified two genetic groups separated by rugged mountainous landscape, which could limit rusa deer movement. However, this finding should be interpreted cautiously, because limited sample sizes, like those in New Zealand in this study, may prevent a full description of the genetic structure or create artificial clusters (Elhaik 2022; Souza et al. 2023). Multiple lineages have been found in the other introduced populations, such as wild boar ( *Sus scrofa* ) (Ishizuka et al. 2025) and red deer ( *Cervus elaphus* ) (Nussey et al. 2006), and as a result, have generated genetic structure in those populations. However, in the case of rusa deer in New Zealand, historical notes indicate a single introduction (Allen 2021). Rusa deer in Te Urewera, New Zealand, mainly occupy lands managed for biodiversity conservation and a management, where the removal of such introduced species is a priority (Allen 2021). Nevertheless, there is increasing interest in rusa deer recreational hunting as trophies (Allen 2021; Nugent 1992), and landowners have sought to retain and translocate rusa deer. As a consequence, apparent escapees from captivity have recently established small populations in several areas throughout the North Island (Allen 2021; Allen et al. 2015; Nugent et al. 2001). It is unlikely that there is gene flow between Australia and New Zealand populations of rusa deer. Although other deer species and large mammals have been reported to be able to swim across the sea (Chen et al. 2025; Ishizuka et al. 2025; Quigley and Moffatt 2014), the distances between our populations (thousands of kilometres) are likely to preclude this. Germplasm can be imported legally to New Zealand from Australia for deer farming. However, rusa deer are rarely farmed in New Zealand (USDA 2023). These contrasting interests may impact the genetic structure of this population, and the acquisition of additional samples would likely enable a more nuanced view of the genetic structure of this introduction.

### 4.2 Loci Under Selection, Potentially Indicating Local Adaptation

Despite reduced genetic diversity in the secondary introductions we studied, these populations appear to be adapting to their new environments. For example, we identified three genes that significantly enriched the GO term ‘regulation of kinase activity’. Protein kinases are enzymes that transfer phosphate groups to proteins and are involved in almost all cellular processes, including cell cycle progression, signal transduction, growth and development, stress response and immune signalling (Bossemeyer 1995). These processes are involved in maintaining metabolic and stress responses and may be relevant for invasive species facing novel environmental conditions. For example, alligator weed ( *Alternanthera philoxeroides* ) that invaded temperate regions from tropical regions showed upregulation of protein kinase genes as has been implicated in the development of cold tolerance (Luo et al. 2020). Reports indicate that rusa deer are sensitive to the temperate New Zealand and Australian ranges, as mortality events among fawns have been recorded during cold‐weather periods (Allen 2021; Mylrea 1991). In addition, the top five outlier SNPs from the BayPass core model were located in or near genes with putative roles for regulation and DNA repair to structural integrity and signalling. *HNRNPA3* is involved in post‐transcriptional regulation (Makeyev et al. 2005). *LRP1B* plays a role in cell communication and lipid homeostasis (Li et al. 2020), while *SGCB* is involved in mechanisms that connect the cytoskeleton to the extracellular matrix and maintain membrane stability (Fougerousse 1998). Additionally, *SHLD2* is involved in DNA repair, contributing to genome stability (Findlay et al. 2018), and *HOMER3* plays a role in intracellular signalling (Duncan et al. 2005). It is plausible that changes to functions such as regulation, DNA repair, structural integrity, and signalling could be relevant to adaptation to novel environments, although further validation would be required.

We found 30 outlier SNPs in the BayPass core model that were also significantly associated with temperature seasonality (Bio4). These SNPs were associated with a set of genes involved in RNA processing (*HNRNPA3*) (Makeyev et al. 2005), membrane/lipid handling and muscle organ development (*LRP1B, SGCB*) (Fougerousse 1998; Li et al. 2020), mitochondrial quality control (*FUNDC2*) (Ma et al. 2019), DNA repair (*SHLD2*) (Findlay et al. 2018), and immune modulation (*LILR*) (Brown et al. 2004). Introduced populations in novel environments experience different host‐pathogen interactions and disease risk (Becvarik et al. 2023; Cohen et al. 2020), possibly creating variable selection on immune modulation genes, like *LILR*. Selection on genes governing transcriptional control, energy balance, and genome maintenance may enhance tolerance to fluctuating temperatures in temperate climates which may recurrently stress cell membranes and cellular metabolism (Lupu et al. 2004; Sokolova et al. 2012; Sorensen et al. 2016). The SNPs associated with temperature seasonality could be important for persistence in environments with variable temperature, despite rusa deer's ancestral tropical morphology that is adapted for sun protection and heat dissipation in their native range (Allen 2021).

Variation in NDVI reflect differences in habitat productivity and resource availability (Borowik et al. 2013), factors that may strongly influence metabolic rate (Auer et al. 2020) and cellular stress (Herring et al. 2011). The differences between native and introduced range habitat productivity may require flexible gene regulation and translational control to optimise metabolism under variable resource availability. We identified 32 SNPs that were both highly differentiated among introduced populations and strongly associated with NDVI. These outlier SNPs were annotated to a set of genes that, in mammals, are involved in gene expression regulation (*HNRNPA3, IER2, DTD2*) (Makeyev et al. 2005), DNA damage repair (*SHLD2*) (Findlay et al. 2018), structural stability (*SGCB, LRPIB, GJC1*) (Fougerousse 1998; Li et al. 2020), metabolic control (*FUNDC2, DAPK2*) (Ber et al. 2015; Ma et al. 2019) and immune modulation (*LILR*) (Brown et al. 2004). Because nutrient limitation can elevate DNA damage (Jin and Wang 2025), selection on DNA repair genes, such as *SHLD2*, which plays a significant role in double‐strand break repair (Findlay et al. 2018), may therefore be relevant for cellular maintenance under resource stress. Furthermore, efficient regulation of energy use and lipid storage may provide metabolic flexibility (Smith et al. 2018; Storlien et al. 2004; Virtue et al. 2018). Putative selection on the *LILR* gene is consistent with a possible role in modulating immune sensitivity to distinct pathogen communities that may arise under different environmental conditions (Bernardo‐Cravo et al. 2020). Together, these associations imply that resource‐driven selection may act on broad physiological pathways that integrate energy balance, cellular maintenance, and immune function, enhancing the rusa deer's ability to adapt to different resources. Similar patterns have been reported in other mammalian studies, where selection linked to environmental conditions appears to act on candidate genes involved in metabolism, cellular maintenance and immune function. For example, white‐footed mouse ( *Peromyscus leucopus* ) populations in urban areas appear to adapt to varying resource availability through genes linked to diet‐related processes (Harris and Munshi‐South 2017). In addition, candidate genes linked to immune response, energy production, and stress response have been identified in invasive raccoon ( *Procyon lotor* ) populations, following exposure to different environmental conditions (Kołodziejczyk et al. 2026).

Given the high false‐positive rates documented for genome‐scan approaches (Sherwin 2022), these signals should be interpreted as putative targets of selection that require further validation, rather than definitive evidence of environment‐specific adaptation. Additional evidence for selection can come from studies of parallel evolution (see next section).

### 4.3 Parallel Evolution

The pairwise BayPass core model analyses using New Caledonia as the baseline identified six shared outlier SNPs in the Australian Illawarra and New Zealand Te Urewera that were annotated to *GAS2*, *REV3L*, *SLC19A1*, *RCBTB1* and *USF3* genes. These genes have diverse but complementary cellular roles in stress response, metabolism and structural regulation. *GAS2* plays a role in linking actin filaments and microtubules, where its coordination drives cell movements, division, and shape changes (Stroud et al. 2014). Selection on *GAS2* may influence how tissues respond to temperature stress (Yang et al. 2017). Additionally, the *REV3L* gene contributes to DNA repair and replication (Actis et al. 2016; Paniagua et al. 2022). In mammals, the *REV3L* gene encodes enzymes that are essential for maintaining genome integrity under replication stress (Martin and Wood 2019). Its detection as a shared outlier may reflect selection on mechanisms related to genomic maintenance under environmental stress. *SLC19A1* mediates folate uptake and cyclic dinucleotide transport, thereby linking metabolic and immune pathways (Zhang et al. 2025). This dual role suggests that putative selection on *SLC19A1* gene could be relevant to how rusa deer populations in both the Australian Illawarra and New Zealand Te Urewerea maintain capacity to cope with variation in dietary resource. *RCBTB1* assists cells to tolerate oxidative stress (Coppieters et al. 2016), and variation in its activity could assist with metabolic and cellular functions when exposed to different environments. *USF3* encodes a transcription factor that promotes bone formation and suppresses bone resorption by regulating WNT16 and RANKL pathways in humans (Ye et al. 2021). Putative selection on the *USF3* gene may indicate how populations balance energy investment between skeletal maintenance and other physiological demands (Suroliya et al. 2024; Wang et al. 2020). The concomitant detection of these genes in both New Caledonia‐Australian Illawarra and New Caledonia‐New Zealand Te Urewera comparisons may indicate parallel evolution, with independently introduced populations derived from the same source showing signatures of selection on shared physiological pathways. This pattern suggests that adaptation in the introduced range may follow some repeatable molecular trajectories, implying a degree of predictability in the evolutionary responses to similar selection regimes. This repeated pattern suggests that these signals are unlikely to be false positives.

This study highlights that even over a short post‐introduction timescale, rusa deer exhibit detectable, shared signals of selection, despite their differentiation at neutral loci. The shared outlier SNPs across the BayPass core and environmental covariate models suggest that local climatic and habitat factors are shaping functional genetic variation in these populations. This underlines how quickly adaptive divergence can occur in newly introduced populations. Although existing literature has documented parallel evolution in introduced populations exposed to similar environments (de Jong et al. 2021; Sherpa et al. 2026; Stuart et al. 2022), the timing of introduction is often not explicitly considered. This is important because differences in time since introduction may affect the relative roles of drift, standing genetic variation, and *de novo* mutations. By focusing on populations introduced from the same source and at the same time, our study provides clearer evidence of parallel evolution.

Furthermore, this study demonstrates that reduced representation sequencing (RRS) remains a crucial tool for detecting signatures of selection (Andrews et al. 2016). RRS is cost‐effective, scalable to large sample sizes and reliable for non‐model species, such as rusa deer. Future work on this system should extend analyses to the native range to assess whether post‐introduction adaptation primarily relies on standing genetic variation or on *de novo* mutations arising after introduction (McGaughran et al. 2025). In the case of rusa deer, because the current native populations are elusive and protected, using preserved historical museum samples is the most effective approach to achieve this. RRS methods have been successfully used for museum samples in other systems to detect signatures of selection (Stuart et al. 2022) and could be used on rusa deer to further investigate evolutionary patterns during invasion.

## Author Contributions

A.N., S.C., W.B.S., and L.A.R. designed the study. P.B. and R.B.A. contributed to sample collection. A.N. performed laboratory work, data analyses and drafted the manuscript. All authors contributed to manuscript revisions and approved the final version of the manuscript.

## Funding

Samples collection in New Caledonia was part of the ‘Lower Jaw Bounty’ project that was primarily funded by the ‘Agence Rurale’, and PROTEGE project that was primarily funded by the European Union (11th EDF) and the ‘Agence Rurale’, with additional contributions from North and South Provinces. The North Province issued the special permit No. 609011‐31/2023/DEPART/JJC 18/08/2023, and a permit was also submitted to the South Province. Sequencing for this study was funded by NSW Department of Primary Industries (Biosecurity and Food Safety and the Special Purpose Pest Management Rate).

## Ethics Statement

The authors have nothing to report.

## Conflicts of Interest

The authors declare no conflicts of interest.

## Supporting information


**Figure S1:** Variation in temperature seasonality across the rusa deer native and introduced ranges. Boxplots show mean and variability in temperature seasonality for the native range distribution (Pairah and Pudyatmoko 2025) and Oceania introduced range sampling points. Introduced populations experience markedly higher temperature seasonality than the native range. Data were sourced from Worldclim (Fick and Hijmans 2017).
**Figure S2:** Variation in Normalised Difference Vegetation Index across rusa deer native and introduced populations. The introduced populations occupy environments that differ in vegetation productivity, with notably lowest NDVI in New Caledonia. Data were downloaded and calculated across sampling points from Sentinel‐2 data using the Copernicus Data Space Ecosystem Browser (https://browser.dataspace.copernicus.eu/). The Native range coordinates were taken from native range distribution (Pairah and Pudyatmoko 2025).
**Figure S3:** Cross‐validation (CV) error values for *K* = 1–10 clusters obtained from the ADMIXTURE analysis of introduced rusa deer populations. CV error decreases sharply from *K* = 1 to 2, and then plateaus, indicating two clusters capture the primary genetic structure.
**Figure S4:** Genome‐wide scan for adaptive divergence and association with temperature seasonality in rusa deer using Baypass.
**Figure S5:** Genome‐wide scan for adaptive divergence and association with NDVI in rusa deer using BayPass.
**Table S1:** List of genes putatively under selection, detected using BayPass core model. The biological function was extracted from GeneCards (Stelzer et al. 2016).
**Table S2:** List of genes putatively under selection and associated with temperature seasonality. Biological function was extracted from Genecards (Stelzer et al. 2016).
**Table S3:** List of genes putatively under selection and associated with NDVI. Biological function was extracted from Genecards (Stelzer et al. 2016).
**Table S4:** List of genes putatively under parallel selection with biological function extracted from Genecards (Stelzer et al. 2016).

## Data Availability

Variant calling data, metadata and code used in this study are available on the Dryad repository: https://doi.org/10.5061/dryad.18931zdc0. Raw DArTseq reads generated in this study are available on NCBI under the BioProject accession PRJNA1499992.
