# Supplementary material for: Two Secondary Introductions From a Shared Bridgehead Population Show Evidence of Divergent and Parallel Selection
Source: Mol Ecol. 2026 Jul 29;35(15):e70495. doi: 10.1111/mec.70495 (PMC13418080; doi:10.1111/mec.70495)
Supplement: Supplementary file 1 — Figure S1: Variation in temperature seasonality across the rusa deer native and introduced ranges. Boxplots show mean and variability in temperature seasonality for the native range distribution (Pairah and Pudyatmoko 2025) and Oceania introduced range sampling points. Introduced populations experience markedly higher temperature seasonality than the native range. Data were sourced from Worldclim (Fick and Hijmans 2017). Figure S2: Variation in Normalised Difference Vegetation Index across rusa deer native and introduced populations. The introduced populations occupy environments that differ in vegetation productivity, with notably lowest NDVI in New Caledonia. Data were downloaded and calculated across sampling points from Sentinel‐2 data using the Copernicus Data Space Ecosystem Browser (https://browser.dataspace.copernicus.eu/). The Native range coordinates were taken from native range distribution (Pairah and Pudyatmoko 2025). Figure S3: Cross‐validation (CV) error values for K = 1–10 clusters obtained from the ADMIXTURE analysis of introduced rusa deer populations. CV error decreases sharply from K = 1 to 2, and then plateaus, indicating two clusters capture the primary genetic structure. Figure S4: Genome‐wide scan for adaptive divergence and association with temperature seasonality in rusa deer using Baypass. Figure S5: Genome‐wide scan for adaptive divergence and association with NDVI in rusa deer using BayPass. Table S1: List of genes putatively under selection, detected using BayPass core model. The biological function was extracted from GeneCards (Stelzer et al. 2016). Table S2: List of genes putatively under selection and associated with temperature seasonality. Biological function was extracted from Genecards (Stelzer et al. 2016). Table S3: List of genes putatively under selection and associated with NDVI. Biological function was extracted from Genecards (Stelzer et al. 2016). Table S4: List of genes putatively under parallel selection with biological [file MEC-35-e70495-s001.docx]

**Supplemental Information for:**

**Two secondary introductions from a shared bridgehead population show evidence of divergent and parallel selection**

**Adi Nugroho^1,2^, Sebastien Comte^1,3,^ Patrick Barrière^4^, Robert B. Allen^5^, William B. Sherwin^1^, Lee A. Rollins^1^**

^1^Evolution & Ecology Research Centre, UNSW Sydney, Sydney, New South Wales, Australia

^2^Department of Bioresources Technology and Veterinary, School of Applied Sciences, Gadjah Mada University, Yogyakarta, Indonesia.

^3^Vertebrae Pest Research Unit, NSW Department of Primary Industries and Regional Development, 1447 Forest Road, Orange, New South Wales, Australia

^4^Agence néo-Calédonienne de la Biodiversité, BP10, Nouvelle-Calédonie

^5^Independent Researcher, Lincoln, New Zealand

**
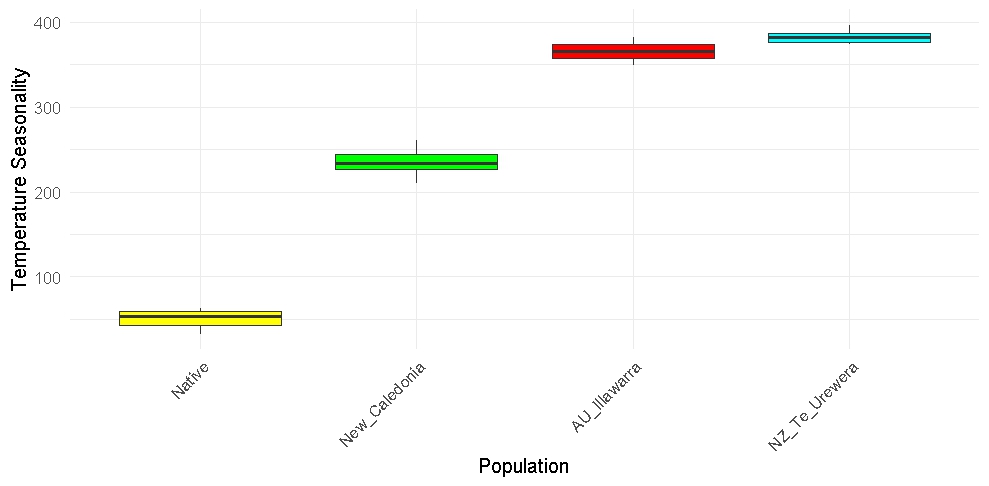
**

**Figure S1**. Variation in temperature seasonality across the rusa deer native and introduced ranges. Boxplots show mean and variability in temperature seasonality for the native range distribution (Pairah & Pudyatmoko, 2025) and Oceania introduced range sampling points. Introduced populations experience markedly higher temperature seasonality than the native range. Data were sourced from Worldclim (Fick & Hijmans, 2017).


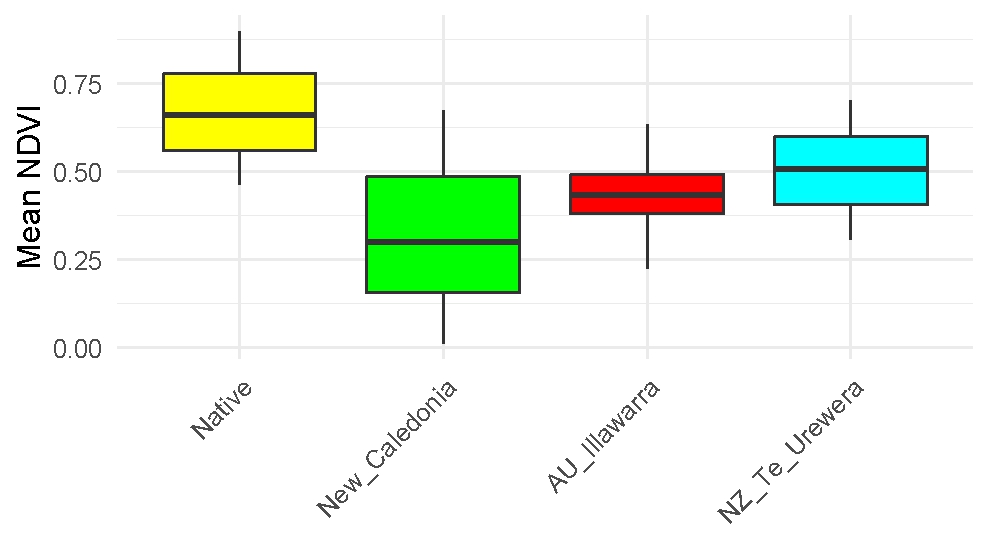


**Figure S2**. Variation in Normalized Difference Vegetation Index across rusa deer native and introduced populations. The introduced populations occupy environments that differ in vegetation productivity, with notably lowest NDVI in New Caledonia. Data were downloaded and calculated across sampling points from Sentinel-2 data using the Copernicus Data Space Ecosystem Browser (<https://browser.dataspace.copernicus.eu/>). The Native range coordinates were taken from native range distribution (Pairah & Pudyatmoko, 2025).


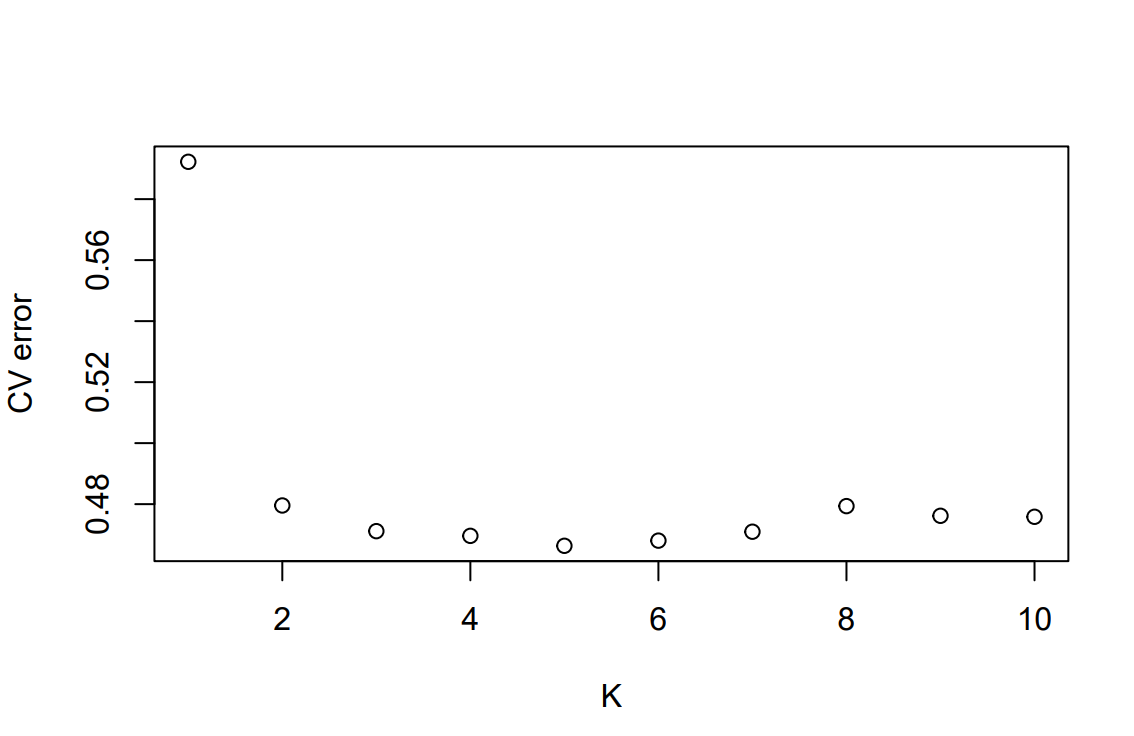


**Figure S3**. Cross-validation (CV) error values for *K*=1-10 clusters obtained from the ADMIXTURE analysis of introduced rusa deer populations. CV error decreases sharply from *K*=1 to 2, and then plateaus, indicating two clusters capture the primary genetic structure.


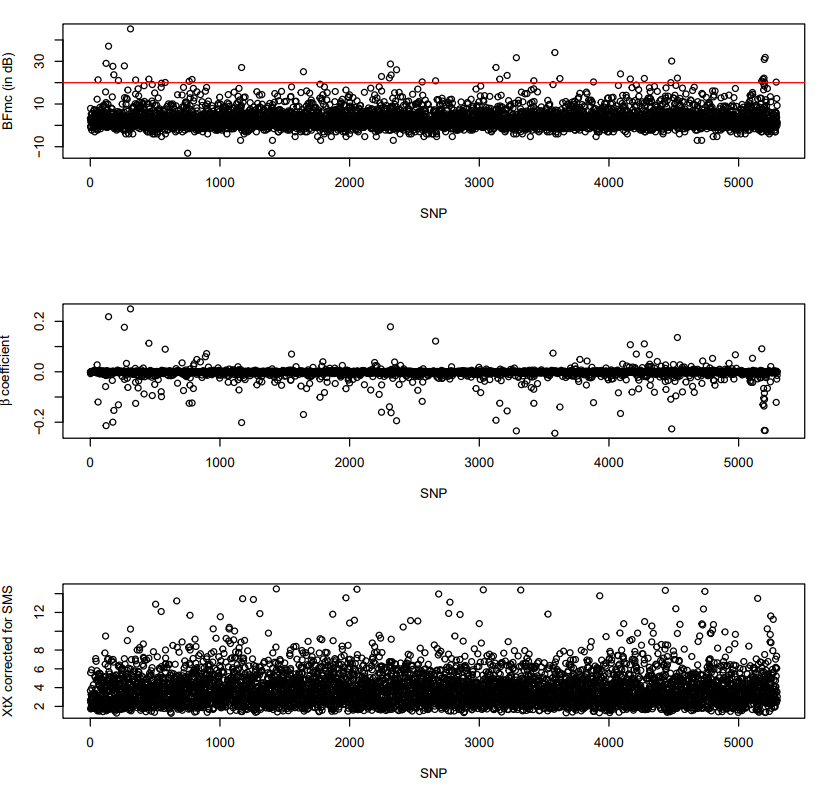


**Figure S4.** Genome-wide scan for adaptive divergence and association with temperature seasonality in rusa deer using Baypass.

1. Bayes factor (BF, in decibels (dB)) for the association between SNP allele frequencies and mean temperature seasonality. The red line at 2- dB denotes the threshold for strong evidence of association
2. Estimated regression coefficients represent the direction and magnitude of allele frequency change with temperature seasonality
3. Posterior mean *XtX* statistic corrected for shared population structure, identifying loci showing elevated genetic differentiation among populations.


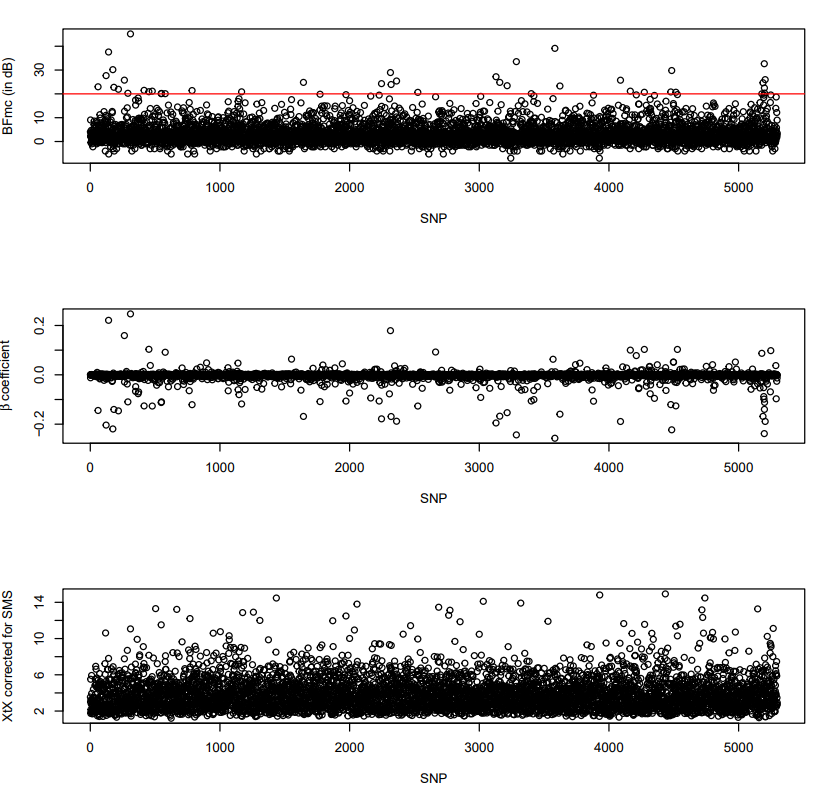


**Figure S5**. Genome-wide scan for adaptive divergence and association with NDVI in rusa deer using BayPass

(A) Bayes factor (BF, in decibels (dB)) for the association between SNP allele frequencies and mean population NDVI. The red line at 20 dB indicates the threshold for strong evidence of association.

(B) Estimated regression coefficients (β) showing the direction and magnitude of allele frequency change with NDVI.

(C) Posterior mean *XtX* statistic corrected for population structure, identifying loci exhibiting elevated genetic differentiation among populations

**Table S1**. List of genes putatively under selection, detected using BayPass core model. The biological function was extracted from GeneCards (Stelzer et al., 2016)

| **No** | **Gene** | **Biological Function** |
| --- | --- | --- |
| 1 | Heterogeneous Nuclear Ribonucleoprotein A3 *(HNRNPA3)* | Enables RNA binding activity. Predicted to be involved in mRNA splicing, via spliceosome. Located in nucleus. Part of catalytic step 2 spliceosome. |
| 2 | LDL Receptor Related Protein 1B *(LRP1B)* | This gene encodes a member of the low density lipoprotein (LDL) receptor family. These receptors play a wide variety of roles in normal cell function and development due to their interactions with multiple ligands. Disruption of this gene has been reported in several types of cancer. |
| 3 | Sarcoglycan Beta *(SGCB)* | This gene encodes a member of the sarcoglycan family. Sarcoglycans are transmembrane components in the dystrophin-glycoprotein complex which help stabilize the muscle fiber membranes and link the muscle cytoskeleton to the extracellular matrix. Mutations in this gene have been associated with limb-girdle muscular dystrophy. |
| 4 | Shieldin Complex Subunit 2 *(SHLD2)* | Involved in negative regulation of double-strand break repair via homologous recombination; positive regulation of double-strand break repair via nonhomologous end joining; and positive regulation of isotype switching. Located in actin cytoskeleton; nucleoplasm; and site of double-strand break. |
| 5 | Homer Scaffold Protein 2 *(HOMER2)* | This gene encodes a member of the homer family of dendritic proteins. Members of this family regulate group 1 metabotrophic glutamate receptor function. The encoded protein is a postsynaptic density scaffolding protein. Alternative splicing results in multiple transcript variant. |
| 6 | Proline Rich 36 *(PRR36)* | This gene encodes a large protein of unknown function that contains internal regions of low complexity sequence. Alternative splicing results in multiple transcript variants. The transcript structure of the protein-coding variant at this locus is conserved between human and mouse. |
| 7 | Natural Cytotoxicity Triggering Receptor 1 *(NCR1)* | Predicted to be involved in immune response-regulating signaling pathway. Predicted to act upstream of or within defense response to virus and detection of virus. Predicted to be located in cell surface. Predicted to be part of SWI/SNF complex. Predicted to be active in plasma membrane. |
| 8 | FA Complementation Group C *(FANCC)* | The Fanconi anemia complementation group (FANC) currently includes FANCA, FANCB, FANCC, FANCD1 (also called BRCA2), FANCD2, FANCE, FANCF, FANCG, FANCI, FANCJ (also called BRIP1), FANCL, FANCM and FANCN (also called PALB2). The previously defined group FANCH is the same as FANCA. Fanconi anemia is a genetically heterogeneous recessive disorder characterized by cytogenetic instability, hypersensitivity to DNA crosslinking agents, increased chromosomal breakage, and defective DNA repair. |
| 9 | FAT Atypical Cadherin 3 *(FAT3)* | Predicted to enable calcium ion binding activity. Predicted to be involved in cell-cell adhesion. Predicted to act upstream of or within amacrine cell differentiation. Predicted to be located in dendrite and plasma membrane. |
| 10 | Gap Junction Protein Gamma 1 *(GJC1)* | This gene is a member of the connexin gene family. The encoded protein is a component of gap junctions, which are composed of arrays of intercellular channels that provide a route for the diffusion of low molecular weight materials from cell to cell. Alternatively spliced transcript variants encoding the same isoform have been described. |
| 11 | Purinergic Receptor P2X 3 *(P2RX3)* | The encoded protein is a subunit of the trimeric P2X3 receptor ion channel which is expressed by sensory or autonomic neurons. A deficiency of the orthologous protein in mice is associated with reduced pain-related behavior and urinary bladder hyporeflexia. |
| 12 | ERBB Receptor Feedback Inhibitor 1 *(ERRFI1)* | ERRFI1 is a cytoplasmic protein whose expression is upregulated with cell growth. It shares significant homology with the protein product of rat gene-33, which is induced during cell stress and mediates cell signaling. |
| 13 | Cilia And Flagella Associated Protein 45 *(CFAP45)* | Enables AMP binding activity. Involved in establishment of left/right asymmetry and flagellated sperm motility. Located in axonemal microtubule and sperm flagellum. Implicated in visceral heterotaxy. |
| 14 | CGRP Receptor Component *(CRCP)* | This gene encodes a membrane protein that functions as part of a receptor complex for a small neuropeptide that increases intracellular cAMP levels. Alternate transcriptional splice variants, encoding different isoforms, have been characterized. |
| 15 | Ring Finger Protein 168 *(RNF168)* | This gene encodes an E3 ubiquitin ligase protein that contains a RING finger, a motif present in a variety of functionally distinct proteins and known to be involved in protein-DNA and protein-protein interactions. The protein is involved in DNA double-strand break (DSB) repair. Mutations in this gene result in Riddle syndrome. |
| 16 | Ectonucleotide Pyrophosphatase/Phosphodiesterase 6 *(ENPP6)* | Enables glycerophosphocholine cholinephosphodiesterase activity. Involved in choline metabolic process and lipid metabolic process. Located in extracellular region and plasma membrane. |
| 17 | LARGE Xylosyl- And Glucuronyltransferase 1 *(LARGE1)* | This gene encodes a member of the N-acetylglucosaminyltransferase gene family. It encodes a glycosyltransferase which participates in glycosylation of alpha-dystroglycan, and may carry out the synthesis of glycoprotein and glycosphingolipid sugar chains. It may also be involved in the addition of a repeated disaccharide unit. The protein encoded by this gene is the glycotransferase that adds the final xylose and glucuronic acid to alpha-dystroglycan and thereby allows alpha-dystroglycan to bind ligands including laminin 211 and neurexin. Mutations in this gene cause several forms of congenital muscular dystrophy characterized by cognitive disability and abnormal glycosylation of alpha-dystroglycan. Alternative splicing of this gene results in multiple transcript variants that encode the same protein. |
| 18 | Microcephalin 1 *(MCPH1)* | This gene encodes a DNA damage response protein. The encoded protein may play a role in G2/M checkpoint arrest via maintenance of inhibitory phosphorylation of cyclin-dependent kinase 1. Mutations in this gene have been associated with primary autosomal recessive microcephaly 1 and premature chromosome condensation syndrome. Alternatively spliced transcript variants have been described. |
| 19 | Erythrocyte Membrane Protein Band 4.1 Like *(EPB41L2)* | Predicted to enable PH domain binding activity; cytoskeletal protein binding activity; and structural molecule activity. Involved in positive regulation of protein localization to cell cortex. Located in several cellular components, including cell cortex; cell junction; and nucleus. |
| 20 | Tetratricopeptide Repeat Domain 31 *(TTC31)* | *TTC31* (Tetratricopeptide Repeat Domain 31) is a Protein Coding gene. Diseases associated with *TTC31* include Adams-Oliver Syndrome. |
| 21 | CD58 Molecule *(CD58)* | This gene encodes a member of the immunoglobulin superfamily. The encoded protein is a ligand of the T lymphocyte CD2 protein, and functions in adhesion and activation of T lymphocytes. The protein is localized to the plasma membrane. Alternatively spliced transcript variants have been described. |
| 22 | Solute Carrier Family 19 Member 1 *(SLC19A1)* | The membrane protein encoded by this gene is a transporter of folate and is involved in the regulation of intracellular concentrations of folate. Three transcript variants encoding different isoforms have been found for this gene. |
| 23 | Ketohexokinase *(KHK)* | This gene encodes ketohexokinase that catalyzes conversion of fructose to fructose-1-phosphate. The product of this gene is the first enzyme with a specialized pathway that catabolizes dietary fructose. Alternatively spliced transcript variants encoding different isoforms have been identified. |
| 24 | TBC1 Domain Family Member 4 *(TBC1D4)* | This gene is a member of the *Tre-2/BUB2/CDC16* domain family. The protein encoded by this gene is a Rab-GTPase-activating protein, and contains two phopshotyrosine-binding domains (*PTB1* and *PTB2*), a calmodulin-binding domain (CBD), a Rab-GTPase domain, and multiple AKT phosphomotifs. This protein is thought to play an important role in glucose homeostasis by regulating the insulin-dependent trafficking of the glucose transporter 4 (GLUT4), important for removing glucose from the bloodstream into skeletal muscle and fat tissues. |
| 25 | Dynactin Associated Protein*(DYNAP)* | Involved in several processes, including activation of protein kinase B activity; cellular response to ergosterol; and positive regulation of cell population proliferation. Located in Golgi apparatus and plasma membrane. |
| 26 | Peptidylglycine Alpha-Amidating Monooxygenase *(PAM)* | This gene encodes a multifunctional protein. The encoded preproprotein is proteolytically processed to generate the mature enzyme. This enzyme includes two domains with distinct catalytic activities, a peptidylglycine alpha-hydroxylating monooxygenase (PHM) domain and a peptidyl-alpha-hydroxyglycine alpha-amidating lyase (PAL) domain. |
| 27 | Zinc Finger Protein 250 *(ZNF250)* | Enables identical protein binding activity and sequence-specific double-stranded DNA binding activity. Predicted to be involved in regulation of transcription by RNA polymerase II. Predicted to be active in nucleus. |
| 28 | Cytochrome P450 Family 2 Subfamily R Member 1 *(CYP2R1)* | This gene encodes a member of the cytochrome P450 superfamily of enzymes. The cytochrome P450 proteins are monooxygenases which catalyze many reactions involved in drug metabolism and synthesis of cholesterol, steroids and other lipids. This enzyme is a microsomal vitamin D hydroxylase that converts vitamin D into the active ligand for the vitamin D receptor. A mutation in this gene has been associated with selective 25-hydroxyvitamin D deficiency. |
| 29 | Divergent Protein Kinase Domain 1B *(DIPK1B)* | This gene encodes a member of the FAM69 family of cysteine-rich type II transmembrane proteins. These proteins localize to the endoplasmic reticulum but their specific functions are unknown. |
| 30 | Protein Phosphatase 1L *(PPM1L)* | The protein encoded by this gene is a magnesium or manganese-requiring phosphatase that is involved in several signaling pathways. The encoded protein downregulates apoptosis signal-regulating kinase 1, a protein that initiates a signaling cascade that leads to apoptosis when cells are subjected to cytotoxic stresses. This protein also is an endoplasmic reticulum transmembrane protein that helps regulate ceramide transport from the endoplasmic reticulum to the Golgi apparatus. Finally, this gene may be involved in adiposity since it is upregulated in adipose tissues. Several transcript variants encoding different isoforms have been found for this gene. |
| 31 | FUN14 Domain Containing 2 *(FUNDC2)* | Enables phosphatidylinositol-3,4,5-trisphosphate binding activity. Involved in intracellular triglyceride homeostasis. |
| 32 | ETS Variant Transcription Factor 6 *(ETV6)* | This gene encodes an ETS family transcription factor. The product of this gene contains two functional domains: a N-terminal pointed (PNT) domain that is involved in protein-protein interactions with itself and other proteins, and a C-terminal DNA-binding domain. Gene knockout studies in mice suggest that it is required for hematopoiesis and maintenance of the developing vascular network. This gene is known to be involved in a large number of chromosomal rearrangements associated with leukemia and congenital fibrosarcoma. |
| 33 | Immediate Early Response 2 *(IER2)* | Predicted to enable DNA binding activity. Involved in cell motility and positive regulation of transcription by RNA polymerase II. Located in cytoplasm and nucleoplasm. Implicated in colorectal cancer and hepatocellular carcinoma. Biomarker of childhood acute myeloid leukemia; colorectal adenocarcinoma; hepatocellular carcinoma; and melanoma. |
| 34 | DNA Fragmentation Factor Subunit Alpha *(DFFA)* | Apoptosis is a cell death process that removes toxic and/or useless cells during mammalian development. The apoptotic process is accompanied by shrinkage and fragmentation of the cells and nuclei and degradation of the chromosomal DNA into nucleosomal units. DNA fragmentation factor (DFF) is a heterodimeric protein of 40-kD (*DFFB*) and 45-kD (*DFFA*) subunits. *DFFA* is the substrate for caspase-3 and triggers DNA fragmentation during apoptosis. DFF becomes activated when *DFFA* is cleaved by caspase-3. The cleaved fragments of *DFFA* dissociate from *DFFB*, the active component of DFF. *DFFB* has been found to trigger both DNA fragmentation and chromatin condensation during apoptosis. Two alternatively spliced transcript variants encoding distinct isoforms have been found for this gene. |
| 35 | NLR Family CARD Domain Containing 5 *(NLRC5)* | This gene encodes a member of the caspase recruitment domain-containing NLR family. This gene plays a role in cytokine response and antiviral immunity through its inhibition of NF-kappa-B activation and negative regulation of type I interferon signaling pathways. |
| 36 | GRB2 Related Adaptor Protein 2 *(GRAP2)* | This gene encodes a member of the GRB2/Sem5/Drk family. This member is an adaptor-like protein involved in leukocyte-specific protein-tyrosine kinase signaling. Like its related family member, GRB2-related adaptor protein (*GRAP*), this protein contains an SH2 domain flanked by two SH3 domains. This protein interacts with other proteins, such as GRB2-associated binding protein 1 (*GAB1*) and the SLP-76 leukocyte protein (*LCP2*), through its SH3 domains. Multiple alternatively spliced transcript variants encoding distinct isoforms have been found for this gene. |
| 37 | Glutamyl-TRNA Synthetase 2 *(EARS2)* | This gene encodes a member of the class I family of aminoacyl-tRNA synthetases. These enzymes play a critical role in protein biosynthesis by charging tRNAs with their cognate amino acids. This protein is encoded by the nuclear genome but is likely to be imported to the mitochondrion where it is thought to catalyze the ligation of glutamate to tRNA molecules. Mutations in this gene have been associated with combined oxidative phosphorylation deficiency 12 (*COXPD12*). Alternative splicing results in multiple transcript variant. |
| 38 | Death Associated Protein Kinase 2*(DAPK2)* | This gene encodes a protein that belongs to the serine/threonine protein kinase family. This protein contains a N-terminal protein kinase domain followed by a conserved calmodulin-binding domain with significant similarity to that of death-associated protein kinase 1 (*DAPK1*), a positive regulator of programmed cell death. Overexpression of this gene was shown to induce cell apoptosis. It uses multiple polyadenylation sites. |
| 39 | Elongation Factor Like GTPase 1 *(EFL1)* | Enables GTPase activity and ribosome binding activity. Involved in GTP metabolic process and cytosolic ribosome assembly. Predicted to be part of ribonucleoprotein complex. Predicted to be active in cytosol. Implicated in Shwachman-Diamond syndrome. |
| 40 | 5-Hydroxytryptamine Receptor 7 *(HTR7)* | The neurotransmitter, serotonin, is thought to play a role in various cognitive and behavioral functions. The serotonin receptor encoded by this gene belongs to the superfamily of G protein-coupled receptors and the gene is a candidate locus for involvement in autistic disorder and other neuropsychiatric disorders. Three splice variants have been identified which encode proteins that differ in the length of their carboxy terminal ends. |
| 41 | Upstream Transcription Factor Family Member 3 *(USF3)* | This gene encodes a large protein that contains a helix-loop-helix domain and a polyglutamine region. A deletion in the polyglutamine region was associated with risk for thyroid carcinoma. |
| 42 | D-Aminoacyl-TRNA Deacylase 2 *(DTD2)* | Enables Ala-tRNA(Thr) deacylase activity. Involved in aminoacyl-tRNA metabolism involved in translational fidelity. Predicted to be active in cytoplasm. |
| 43 | Leukocyte immunoglobulin-like receptor  *(LILR)* | Regulation of inflammation, immune tolerance cell differentiation and nervous system plasticity. |

**Table S2**. List of genes putatively under selection and associated with temperature seasonality. Biological function was extracted from Genecards (Stelzer et al., 2016)

| **No** | **Gene** | **Biological Function** |
| --- | --- | --- |
| 1 | Heterogeneous Nuclear Ribonucleoprotein A3 *(HNRNPA3)* | Enables RNA binding activity. Predicted to be involved in mRNA splicing, via spliceosome. Located in nucleus. Part of catalytic step 2 spliceosome. |
| 2 | LDL Receptor Related Protein 1B *(LRP1B)* | This gene encodes a member of the low density lipoprotein (LDL) receptor family. These receptors play a wide variety of roles in normal cell function and development due to their interactions with multiple ligands. Disruption of this gene has been reported in several types of cancer. |
| 3 | Sarcoglycan Beta *(SGCB)* | This gene encodes a member of the sarcoglycan family. Sarcoglycans are transmembrane components in the dystrophin-glycoprotein complex which help stabilize the muscle fiber membranes and link the muscle cytoskeleton to the extracellular matrix. Mutations in this gene have been associated with limb-girdle muscular dystrophy. |
| 4 | Shieldin Complex Subunit 2 *(SHLD2)* | Involved in negative regulation of double-strand break repair via homologous recombination; positive regulation of double-strand break repair via nonhomologous end joining; and positive regulation of isotype switching. Located in actin cytoskeleton; nucleoplasm; and site of double-strand break. |
| 5 | Proline-rich protein 36 *(PRR36)* | This gene encodes a large protein of unknown function that contains internal regions of low complexity sequence. Alternative splicing results in multiple transcript variants. The transcript structure of the protein-coding variant at this locus is conserved between human and mouse. |
| 6 | Tetratricopeptide Repeat Domain 31 *(TTC31)* | *TTC31* (Tetratricopeptide Repeat Domain 31) is a Protein Coding gene. Diseases associated with *TTC31* include Adams-Oliver Syndrome. |
| 7 | Divergent Protein Kinase Domain 1B *(DIPK1B)* | This gene encodes a member of the FAM69 family of cysteine-rich type II transmembrane proteins. These proteins localize to the endoplasmic reticulum but their specific functions are unknown. |
| 8 | FUN14 Domain Containing 2 *(FUNDC2)* | Enables phosphatidylinositol-3,4,5-trisphosphate binding activity. Involved in intracellular triglyceride homeostasis. |
| 9 | Immediate Early Response 2 *(IER2)* | Predicted to enable DNA binding activity. Involved in cell motility and positive regulation of transcription by RNA polymerase II. Located in cytoplasm and nucleoplasm. Implicated in colorectal cancer and hepatocellular carcinoma. Biomarker of childhood acute myeloid leukemia; colorectal adenocarcinoma; hepatocellular carcinoma; and melanoma. |
| 10 | D-Aminoacyl-TRNA Deacylase 2 *(DTD2)* | Enables Ala-tRNA(Thr) deacylase activity. Involved in aminoacyl-tRNA metabolism involved in translational fidelity. Predicted to be active in cytoplasm. |
| 11 | Leukocyte immunoglobulin-like receptor  *(LILR)* | Regulation of inflammation, immune tolerance cell differentiation and nervous system plasticity. |

**Table S3.** List of genes putatively under selection and associated with NDVI. Biological function was extracted from Genecards (Stelzer et al., 2016)

| **No** | **Gene** | **Biological Function** |
| --- | --- | --- |
| 1 | Heterogeneous Nuclear Ribonucleoprotein A3 *(HNRNPA3)* | Enables RNA binding activity. Predicted to be involved in mRNA splicing, via spliceosome. Located in nucleus. Part of catalytic step 2 spliceosome. |
| 2 | LDL Receptor Related Protein 1B *(LRP1B)* | This gene encodes a member of the low density lipoprotein (LDL) receptor family. These receptors play a wide variety of roles in normal cell function and development due to their interactions with multiple ligands. |
| 3 | Sarcoglycan Beta *(SGCB)* | This gene encodes a member of the sarcoglycan family. Sarcoglycans are transmembrane components in the dystrophin-glycoprotein complex which help stabilize the muscle fiber membranes and link the muscle cytoskeleton to the extracellular matrix. Mutations in this gene have been associated with limb-girdle muscular dystrophy. |
| 4 | Shieldin Complex Subunit 2 *(SHLD2)* | Involved in negative regulation of double-strand break repair via homologous recombination; positive regulation of double-strand break repair via nonhomologous end joining; and positive regulation of isotype switching. Located in actin cytoskeleton; nucleoplasm; and site of double-strand break. |
| 5 | Proline Rich 36 *(PRR36)* | This gene encodes a large protein of unknown function that contains internal regions of low complexity sequence. Alternative splicing results in multiple transcript variants. The transcript structure of the protein-coding variant at this locus is conserved between human and mouse. |
| 6 | Gap Junction Protein Gamma 1 *(GJC1)* | This gene is a member of the connexin gene family. The encoded protein is a component of gap junctions, which are composed of arrays of intercellular channels that provide a route for the diffusion of low molecular weight materials from cell to cell. Alternatively spliced transcript variants encoding the same isoform have been described. |
| 7 | Divergent Protein Kinase Domain 1B *(DIPK1B)* | This gene encodes a member of the FAM69 family of cysteine-rich type II transmembrane proteins. These proteins localize to the endoplasmic reticulum but their specific functions are unknown. |
| 8 | FUN14 Domain Containing 2 *(FUNDC2)* | Enables phosphatidylinositol-3,4,5-trisphosphate binding activity. Involved in intracellular triglyceride homeostasis. |
| 9 | Immediate Early Response 2 *(IER2)* | Predicted to enable DNA binding activity. Involved in cell motility and positive regulation of transcription by RNA polymerase II. Located in cytoplasm and nucleoplasm. Implicated in colorectal cancer and hepatocellular carcinoma. Biomarker of childhood acute myeloid leukemia; colorectal adenocarcinoma; hepatocellular carcinoma; and melanoma. |
| 10 | Death Associated Protein Kinase 2 *(DAPK2)* | This gene encodes a protein that belongs to the serine/threonine protein kinase family. This protein contains a N-terminal protein kinase domain followed by a conserved calmodulin-binding domain with significant similarity to that of death-associated protein kinase 1 (DAPK1), a positive regulator of programmed cell death. Overexpression of this gene was shown to induce cell apoptosis. It uses multiple polyadenylation sites. |
| 11 | D-Aminoacyl-TRNA Deacylase 2 *(DTD2)* | Enables Ala-tRNA(Thr) deacylase activity. Involved in aminoacyl-tRNA metabolism involved in translational fidelity. Predicted to be active in cytoplasm. |
| 12 | Leukocyte immunoglobulin-like receptor  *(LILR)* | Regulation of inflammation, immune tolerance cell differentiation and nervous system plasticity. |

**Table S4**. List of genes putatively under parallel selection with biological function extracted from Genecards (Stelzer et al., 2016)

| **No** | **Gene** | **Biological Function** |
| --- | --- | --- |
| 1 | Growth Arrest Specific 2 *(GAS2)* | The protein encoded by this gene is a caspase-3 substrate that plays a role in regulating microfilament and cell shape changes during apoptosis. It can also modulate cell susceptibility to p53-dependent apoptosis by inhibiting calpain activity. Alternative splicing results in multiple transcript variants. |
| 2 | REV3 Like, DNA Directed Polymerase Zeta Catalytic Subunit *(REV3L)* | The protein encoded by this gene represents the catalytic subunit of DNA polymerase zeta, which functions in translesion DNA synthesis. The encoded protein can be found in mitochondria, where it protects DNA from damage. Defects in this gene are a cause of Mobius syndrome. |
| 3 | Solute Carrier Family 19 Member 1 *(SLC19A1)* | The membrane protein encoded by this gene is a transporter of folate and is involved in the regulation of intracellular concentrations of folate. Three transcript variants encoding different isoforms have been found for this gene. |
| 4 | RCC1 And BTB Domain Containing Protein 1 *(RCBTB1)* | This gene encodes a protein with an N-terminal RCC1 domain and a C-terminal BTB (broad complex, tramtrack and bric-a-brac) domain. In rat, over-expression of this gene in vascular smooth muscle cells induced cellular hypertrophy. |
| 5 | Upstream Transcription Factor Family Member 3 *(USF3)* | This gene encodes a large protein that contains a helix-loop-helix domain and a polyglutamine region. A deletion in the polyglutamine region was associated with risk for thyroid carcinoma. |

**References**

Fick, S. E., & Hijmans, R. J. (2017). WorldClim 2: new 1‐km spatial resolution climate surfaces for global land areas. *International Journal of Climatology*, *37*(12), 4302-4315. <https://doi.org/10.1002/joc.5086>

Pairah, P., & Pudyatmoko, S. (2025). Javan Deer Rusa timorensis (de Blainville, 1822). In M. F. Melletti, Stefano (Ed.), *Deer of The World*. Springer <https://doi.org/https://doi.org/10.1007/978-3-031-17756-9>

Stelzer, G., Rosen, N., Plaschkes, I., Zimmerman, S., Twik, M., Fishilevich, S., Stein, T. I., Nudel, R., Lieder, I., Mazor, Y., Kaplan, S., Dahary, D., Warshawsky, D., Guan-Golan, Y., Kohn, A., Rappaport, N., Safran, M., & Lancet, D. (2016). The GeneCards Suite: From Gene Data Mining to Disease Genome Sequence Analyses. *Curr Protoc Bioinformatics*, *54*, 1 30 31-31 30 33. <https://doi.org/10.1002/cpbi.5>
